# Water sorption of gingiva-shade composites and its influence on color stability

**DOI:** 10.3389/fdmed.2025.1671386

**Published:** 2025-09-23

**Authors:** Shivaughn M. Marchan, Kelee Bascombe, Samiyah Suliman, Reisha N. Rafeek

**Affiliations:** Unit of Restorative Dentistry, School of Dentistry, Faculty of Medical Sciences, The University of the West Indies, St. Augustine, Trinidad and Tobago

**Keywords:** gingiva-shade composites, water sorption, color stability, giomer, Pearson's correlation

## Abstract

**Background:**

This study aimed to ascertain the water sorption of three gingival shade esthetic materials and understand the correlation between water sorption and color change when immersed in common food colorants.

**Methods:**

Disc-shaped composites were fabricated and subjected to immersion in distilled water, followed by desiccation to ascertain water sorption values. Simultaneously, similarly shaped specimens were fabricated, measured for total color using a spectrophotometer before and after immersion in various colorant solutions. Pearson's correlation was used to ascertain if there was a positive linear relationship between values for water sorption and change in total color.

**Results:**

There was a statistically significant difference in mean water sorption data between the tested composites (*p* = 0.02, *F* value = 5.6). No correlation coefficients between water sorption and any tested composites in any colorant solutions were statistically significant (*p* ≥ 0.05), with the exception of PermaFlow Pink immersed in coffee (*r* = −0.635, *p* = 0.05).

**Conclusions:**

All the tested composites exhibited favorable values for water sorption^.^ Over the studied period, water immersion produced a negligible change in color among all the tested specimens. All composites exhibited changes in color when exposed to food colorants with the largest relative change occurring for immersion in curry. There was no positive correlation between water sorption values and change in color.

## Introduction

Polymerized dental composite absorbs water primarily as a function of its resin matrix (1). Water sorption describes the net movement of water into the composite, which can influence mechanical strength, color stability, and the bond strengths to the tooth substrate (1). The ISO standard for dental composite limits water sorption to 40 µg/mm^(3)^ (2). Absorption levels above this value cause chemical deterioration with release of uncured residual monomer, filler degradation, and hydrolysis of the oxane bonds between matrix and filler (3). Mechanical properties of dental composites vary under dry and wet conditions, implicating an effect of water sorption on material properties (4).

Research into tooth-colored giomers has demonstrated greater values for water sorption compared with dental composites (5, 6). Giomers are known to release fluoride due to their unique filler chemistry of surface pre-reacted glass particles. Fluoride release is influenced by the ability of the material to absorb water, which may explain, in part, the greater observed values for water sorption (6). Water movement into the material to facilitate fluoride release must be mitigated, as excessive water sorption into the material causes degradation (6).

The water sorption of dental composite is influenced by several factors, including the proportion of hydrophilic to hydrophobic monomers, cross-linking of monomer chains, filler loading, and porosities within the polymerized dental composite (7–9). The literature describes a correlation between water sorption and color stability for tooth-colored-giomers and dental composites (10). Gingiva colored composites and giomers (GCRBC) like tooth-colored composites contain a resin matrix of principal and diluent monomers, silane-coated inorganic filler particles, photo-initiators, and various tints and pigments (11). GCRBCs additionally contain red dyes, inclusive of red iron oxide (FDA dye no 5595) and disodium salts of sulfonic acid (FDA dye no 40) to simulate gingival color (12). These pigments vary in concentration from 0.005% to 0.75% relative to the resin weight component (13). Pigments, as color modifiers, have been shown to influence the degree of conversion of composite resins and water sorption, with effects on the color stability of GCRBCs (14). Given that these materials can have up to 0.75% by weight of included pigment modifiers, water sorption and subsequent color stability can be negatively influenced (12, 14).

GCRBCs can be used to optimize or maintain esthetic ratios of pink to white when non-carious cervical lesions need to be restored or when periodontal surgery to correct recession defects is not indicated (15). Recent work has demonstrated large changes in the total color of GCRBC compared to a tooth-colored nanohybrid when subjected to common food colorants such as coffee and curry, with the authors postulating that color change in these composites is related to the relative surface condition of the materials (15). This study aimed to ascertain the water sorption of three gingiva shade esthetic materials and understand the correlation between water sorption and the color stability of these materials when immersed in common food colorants. The null hypothesis stated that there would be no difference in mean water sorption values among any of the tested materials and no linear relationship between water sorption and color stability for any of the tested materials.

## Materials and methods

**Figure 1:**
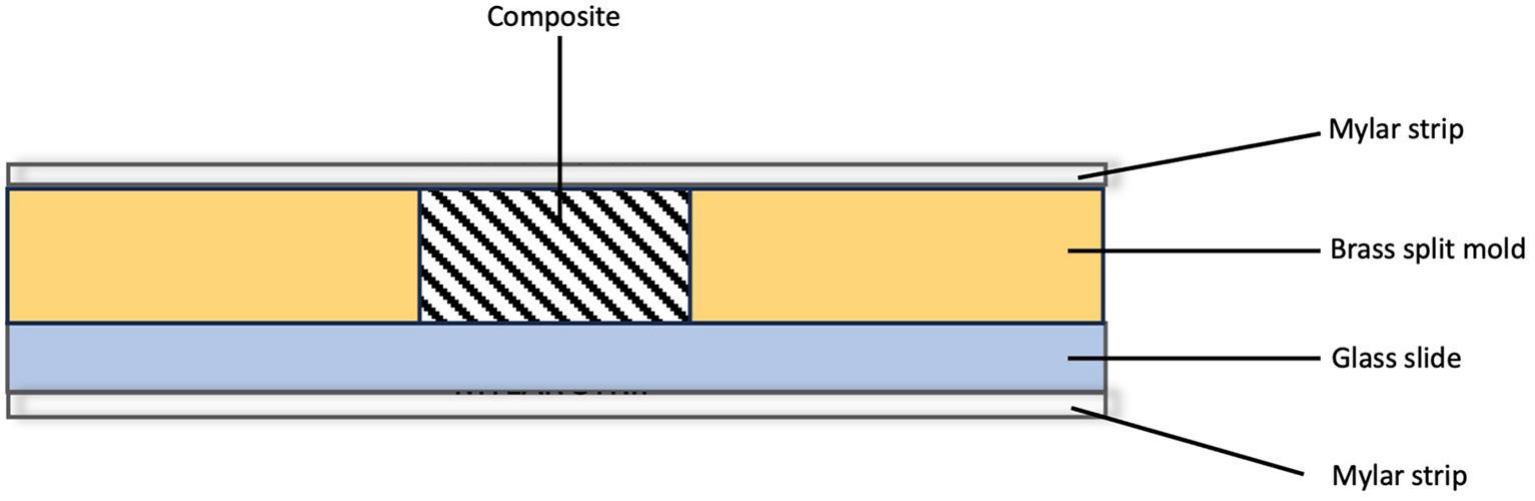
Diagrammatic representation of specimen preparation.

The materials used in this study are included in Table 1. Disc-shaped specimens measuring 7 mm in internal diameter and 2 mm in thickness. were fabricated using a brass split-ring mold. The bottom surface of the mold consisted of a Mylar strip mounted on a glass slide. Composite was placed into the split mold, a second mylar strip was placed, and manual pressure was applied using a second glass slide. The glass slide was removed and the composite polymerized using an LED light (Valo, Ultradent Products, UT, USA) with a wavelength between 385 and 515 nm and intensity of 970 mW/cm^(2)^ (Figure 1). All specimens were cured according to the manufacturer's instructions. Excess material was carefully trimmed using a scalpel. Each sample was then inspected for any voids or surface irregularities.

**Table 1:** Composites used in this study.

Composite	Manufacturer details
Beautifil Pink	Shofu Corporation, San Marcos, CA, USA
PermaFlo Pink	Ultradent, South Jordan, UT, USA
Amaris Gingiva	Voco America Inc, SC, USA
Nanohybrid Universal (A3)	3M, St. Paul, MN, USA

A power analysis determined that 10 samples per material were required for the assessment of water sorption, as listed in Table 1. The power analysis was calculated using an effect size of 0.4, an alpha level of 0.05, a power of 0.8, and a total of five groups for each of the four tested composites for the color stability measurements. The samples were initially stored dry in a light-proof container before immersion in distilled water. One day later, the specimens were weighed (m_0_) on an analytical beam balance with an accuracy of 0.0001 g (Veritas, H&C Weighing Systems). Immediately following weighing, each specimen of each composite was immersed in individual wells containing 100 ml of distilled water. These individual wells were then placed in an incubator set at 37°C. Following immersion, specimens were weighed at 2, 4, and 6 h on day 1. Specimens were then weighed on days 2 and 3 and weeks 1 and 2. The protocol for weighing the specimens involved taking the specimen from the individual well and gently blotting the top and bottom of each specimen with clean absorbent laboratory tissue (Kimtech Science Laboratory Tissues, Kimberly-Clarke, USA). The specimen was weighed on the analytical balance, which was placed on an anti-vibration pad (Rice Lake Weighing Systems, USA) and had a built-in draft shield.

**Figure 2:**
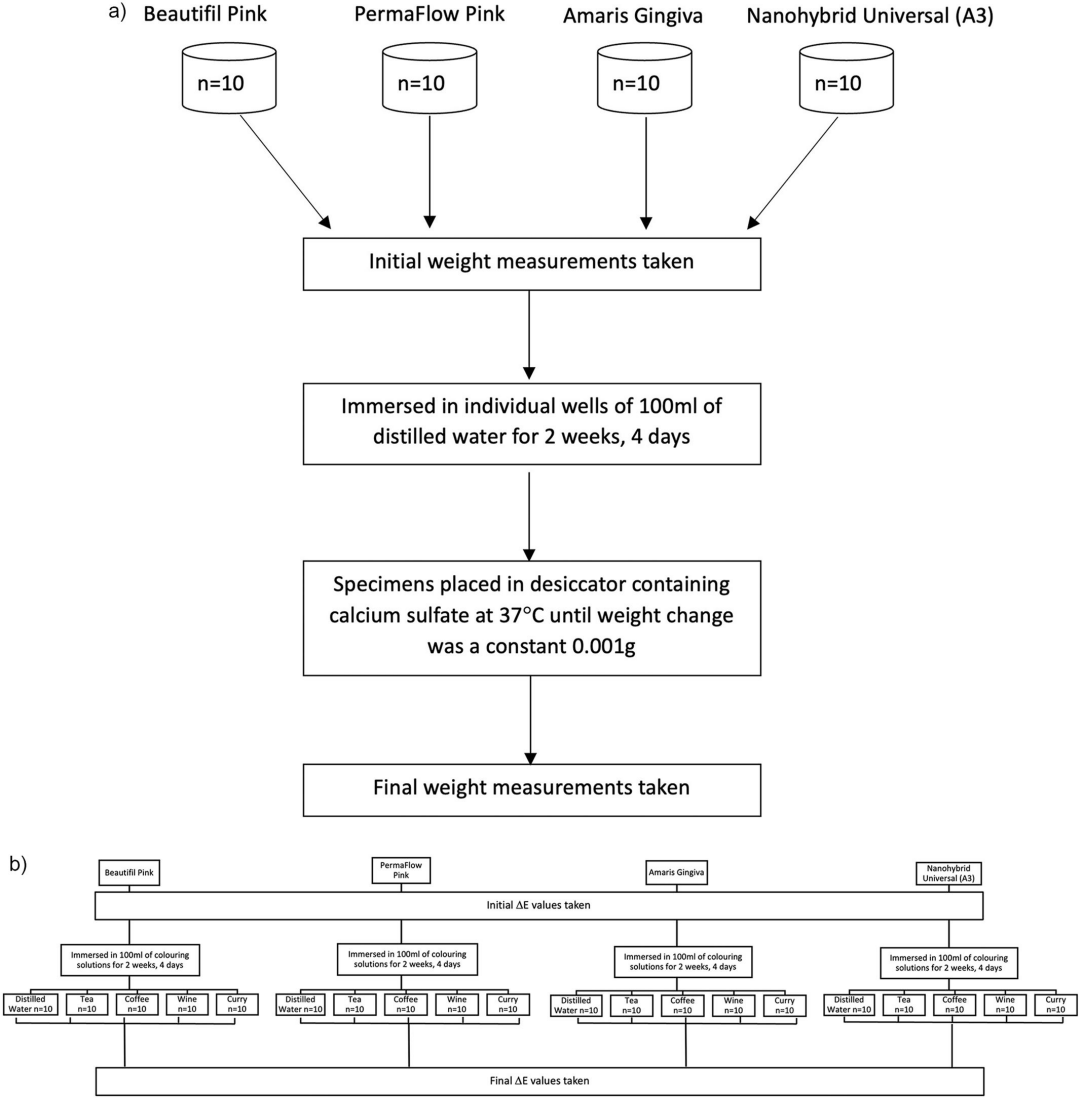
**(a)** Flowchart of water sorption protocol. **(b)** Flowchart showing color measurement protocol.

Immediately after weighing, each specimen was re-immersed in water and returned to the incubator. The time each specimen spent out of the water during weighing did not exceed 30 s. Once the weight change between measurements was between 0.001 g, the specimens were considered to have reached equilibrium in terms of water sorption (Figure 2a). The specimens were then placed in a desiccator containing calcium sulfate at 37°C until the weight change between measurements was a constant 0.001 g. Finally, the specimens were placed in a dry heat oven at 60°C for 24 h before the final weight measurements were taken (m_2_). Water sorption was calculated using the formula: (m_0_-m_2_)/v,

where m_0_ is the mass before immersion, m_2_ is the mass after immersion and desiccation, and v is the volume of the disc-shaped specimen (π. *r*^2^.h), where r is the radius of the specimen (3.5 mm) and h is the height (thickness) of the specimen.

Simultaneously, 50 specimens of each material were fabricated using the same brass split mold. The composites were polymerized with a VALO curing light (Ultradent, USA) in a continuous curing mode, with an output of 970 mW/cm^2^ for 20 s in accordance with the individual manufacturer's instructions. Composite flash was removed from the periphery with a 15-scalpel blade, and the surfaces were inspected for any visible defects. Immediately after preparation, the specimens were stored in a black, light-proof container at room temperature to prevent further polymerization due to ambient light. Specimens were initially bagged and numbered, and a randomizer application (Randomizer.org) was used to randomly divide the specimens into five groups: One control group and 4 experimental groups of food colorants: coffee, curry, tea, and wine (Figure 2b). The food colorant groups were as follows:

Group 1: Distilled water, Group 2: 40 ml of red wine (Carlo Rossi Red^TM^), Group 3: 40 ml of a heterogeneous solution of curry (Ground Massala, Chief Brand Curry, Trinidad ^TM^) in a ratio of 15 ml of curry powder to 100 ml of distilled water. Group 4: 40 ml solution of black tea (Lipton Yellow Lab Tea^TM^) in a ratio of 1 tea bag to 100 ml of distilled water, Group 5: 40 ml of coffee (Nescafe Gold Blend) in a ratio of 15 mg coffee granules to 100 ml water. The protocol for the preparation of the colorant solutions was in keeping with previously published work that explored the color stability of GCRBCs (15).

Before immersion, baseline color measurements were taken for all the composite samples within the CIE (Commission Internationale de l'Eclairage) L*a*b* color space using a CI 7600 X-rite (Pantone LLC, MI, USA) desktop digital spectrophotometer with a corrected D65 light-source and an aperture distance of 6 mm. For each tested specimen, four measurements were taken for each color parameter, and the mean was calculated. The X-rite digital spectrophotometer was calibrated against a white ceramic reference and twelve BCRA(CERAM Research Ceramics Standards**)** Series II Color tiles before color measurements were taken. The Spectrophotometer was checked for repeatability and inter-instrument agreement with the average performance for inter-instrument agreement with the spectral component included being ΔE = 0.15 (test limit: ΔE ≤ 0.30) and white repeatability performance of Max RMS being ΔE = 0.008 (test limit: Max RMS ΔE ≤ 0.030).

Total color (ΔE) was automatically calculated for each specimen using the following formula:

$$\Delta E\;=\;[{\left(\Delta L*\right)}^{2}\;+\;{\left(\Delta a*\right)}^{2}\;+\;{\left(\Delta b*\right)}^{2}]$$

Immersion of composite specimens in the respective food colorants occurred over two weeks and four days in a lightproof container in a dark storage cupboard at room temperature, with solutions being changed every 2 days. The length of time in the colorant solutions coincided with the length of time for the water sorption measurements (immersion and desiccation). At the end of the immersion period, composite specimens were removed, carefully washed with distilled water, and blotted dry before post-immersion color measurements could take place. Change in total color was calculated using the formula:

$$\Delta E_{\left(\mathrm{pre}\text{-}\mathrm{immersion}\right)}-\;\Delta E_{\left(\mathrm{post}\text{-}\mathrm{immersion}\right)}=\Delta E_{\left(\mathrm{color}\;\mathrm{change}\right)}$$

A one-way analysis of variance (ANOVA) was used to determine statistically significant differences in the mean values for water sorption and color change for each of the tested composites in the various food colorants. *Post-hoc* Bonferroni tests, at an alpha level of 0.05, were used for multiple comparisons of water sorption and color change for each tested material in the various colorant solutions. Pearson's correlation was used to ascertain if there was a positive linear relationship between values for water sorption and change in ΔE. A Shapiro–Wilk test determined that the dependent variables were normally distributed before the use of the parametric ANOVA and Pearson's Correlation.

## Results

Mean water sorption data for the tested GCRBCs are presented in Table 2. The water sorption data for two specimens of Amaris presented as outliers compared with the remaining eight specimens. The mean values of the remaining eight specimens were substituted for these outliers to facilitate statistical analyses. There were statistically significant differences in mean water sorption data between the tested composites (*p* = 0.02, *F* value = 5.6). *Post-hoc* Bonferroni demonstrated significant differences between Amaris Gingiva and Permaflow Pink (*p* = 0.001). Mean color change data (change in ΔE) are presented in Table 3 together with significances. There was no statistically significant difference in the color change of any of the tested composites because of immersion in distilled water; however, there were mixed results between the materials for the various colorant solutions. Correlation coefficients, together with significances, are presented in Table 4. No correlation coefficients, except PermaFlow immersed in a coffee solution, between water sorption and any tested composites in any colorant solutions were statistically significant (*p* ≥ 0.05).

**Table 2:** Means (standard deviations) water sorption values of tested composites.

Composite	Water Sorption (µg/mm^3^) Mean ± SD
Beautifil Pink	11.169 ± 3.58^a^
PermaFlo Pink	15.714 ± 5.457^A^
Amaris Gingiva	8.442 ± 3.353^a^
Nanohybrid Universal (A3)	11.429 ± 2.654^a^

Statistically significant differences (*p* ≤ 0.05), determined by *post-hoc* analysis, between groups of composites for water sorption are denoted by different capital and common letters, *p* = 0.02.

**Table 3:** Mean change in ΔE from baseline, after immersion in various food colorants.

Composite	Mean Change in ΔE ± SD				
	Water	Wine	Tea	Coffee	Curry
Beautifil Pink	0.09 **±** 0.29^a^	0.54 **±** 0.71^b^	1.74 **±** 0.83^C^	2.86 **±** 0.83^d^	13.73 **±** 4.38^E^
PermaFlo Pink	0.39 **±** 0.53^a^	0.51 **±** 0.20^B^	1.01 **±** 0.42^c^	3.17 **±** 1.02^D^	18.69 **±** 2.06^e^
Amaris Gingiva	0.16 **±** 0.23^a^	0.95 **±** 1.5^b^	1.65 **±** 0.95^C^	2.66 **±** 0.57^d^	22.34 **±** 6.05^e^
Universal nanohybrid	0.14 **±** 0.29^a^	1.4 **±** 0.78^b^	0.43 **±** 0.87^c^	1.81 **±** 1.10^d^	16.32 **±** 6.23^e^

Statistically significant differences (*p* ≤ 0.05), determined by *post-hoc* analysis, between groups of composites for each food colorant are denoted by different capital and common superscript letters.

**Table 4:** Correlations between water sorption and change in ΔE with each of the colorant solutions.

Composite	Correlation coefficient with p-values	Colorant solutions				
		Water	Tea	Wine	Coffee	Curry
Beautifil Pink	Pearson Correlation	−0.628	0.337	0.448	−0.111	−0.297
	*P* value	0.052	0.341	0.194	0.760	0.404
PermaFlo Pink	Pearson Correlation	0.430	−0.022	−0.346	−0.635	−0.090
	*P* value	0.214	0.952	0.328	0.050	0.805
Amaris Gingiva	Pearson Correlation	−0.416	−0.028	0.105	0.052	0.367
	*P* value	0.232	0.939	0.773	0.886	0.298
Nanohybrid	Pearson Correlation	−0.031	−0.629	−0.600	−0.562	0.271
	*P* value	0.933	0.051	0.067	0.091	0.448

## Discussion

All values for water sorption were well below the upper limit of 40 µg/mm^3^ set by the ISO for acceptable water sorption (2). The values for water sorption attained in this study would mean minimal chemical and physical degradation of the tested composites as a result of placement in a biologically moist environment (3). The relative values for water sorption were higher compared to previously conducted water sorption research on giomers and tooth colored resins, but these results demonstrate a key challenge with this type of work when the sizes of composite samples are not standardized across studies, thus making direct comparisons on values of water sorption difficult (6).

The water sorption values for PermaFlo Pink were greater than for the other tested composites and statistically higher than those of Amaris Gingiva. This could be attributed to the classification of this material as a flowable composite with lower filler loading by weight. Composite resins with higher filler loading have demonstrated lower water sorption values, with larger filler loading of the resin matrix reducing available space for water uptake (2, 8, 16).

PermaFlo Pink, according to the manufacturer's details, contains 0.3% sodium monofluorophosphate by weight. Fluoride added as salts, including sodium monofluorophosphate, is unevenly distributed throughout the resin matrix and is released quickly when immersed in water (17). This early fluoride release can result in voids, which influence net water uptake by the resin composite. Research has shown that materials with added fluoride salts tend to have lower mechanical properties, influenced in part by water sorption after early release of fluoride (17, 18).

The relative proportions of the hydrophilic and hydrophobic resins within the resin matrix can influence water sorption (19). According to the manufacturer's details, PermaFlo Pink contains up to 20% by weight of Triethylene Glycol Dimethacrylate (TEGDMA). TEGDMA is a low-viscosity, diluent, hydrophilic monomer used as a cross-linking agent in many dental composite formulations. The presence of hydrophilic monomers within the resin matrix can influence the net flow of water into the polymerized sample, as evidenced by research by Ito et al, who demonstrated negligible water sorption with the least hydrophilic experimental formulas of dental composite (20). The lower relative proportions to TEGDM, as listed by the manufacturers, for the other GCRBCs varied from <2.5% (Amaris Gingiva) to 1%–10% (Beautifil Gingiva II) by percentage weight.

This study used samples of composite material, which were simultaneously prepared and independently tested for water sorption and color stability. While it may be argued that separate specimens cannot be directly compared for correlation, the exact treatment of the specimens regarding volumetric dimensions, the amount of liquid in which the specimens were immersed for both the water sorption and color measurements, and the length of time in liquid helped standardize the experimental conditions for correlational analysis.

Immersion in the distilled water colorant solutions for 2 weeks and 4 days translated into the consumption of approximately 2,600 beverages, assuming a 10-min consumption time for each beverage. Immersion in all colorant solutions produced changes in ΔE, with immersion in water causing the smallest shift in ΔE values among all the tested materials. The nanohybrid produced the smallest relative change in ΔE in all the colorant solutions. Immersion in a solution of curry produced the largest change in ΔE for all the tested materials. This concurs with the findings of recent research, which showed an unacceptable change in the color of gingival shade composites when immersed in a suspension of curry powder (15). The yellow color of curry is derived from curcumin, a polyphenolic hydrophobic phytopigment, which is easily adsorbed onto the surface of dental composite materials (21).

In the present study, the change in ΔE showed no positive correlation with water sorption among the tested materials, given either negative correlation values or low correlations that were not statistically significant between water sorption and the change in ΔE when immersed in the various colorant solutions.

Given that the relative change in ΔE was not positively correlated with water sorption values, the authors postulated another cause for the change in ΔE for both the tooth- and gingiva-colored composites. Even the statistically significant negative correlation between water sorption and change in color (*r* = −0.635, *p* = 0.050) noted with Permaflow Pink immersed in coffee would indicate another mechanism besides the net movement of water into the GCRBC in causing color change. Extrinsic staining or adsorption of stain onto the surface of the composites could have accounted for the staining noted with these materials. Surface finish of composite materials is known to influence color change, with rougher composite surfaces demonstrating larger and clinically perceptible changes in color (22). Previous research on the color stability of the gingiva-colored composites revealed Amaris Gingiva to have visually rougher surfaces compared to similar composites after polishing (15). This, in part, could have explained the statistically significant color change when immersed in tea and curry solutions when compared to the nanohybrid and Beautifil Pink, respectively. Lack of surface topographical assessment of the composite samples is a major limitation of this work. Quantitative profilometric assessment and qualitative assessment using scanning electron microscopy are planned to ascertain whether the surface roughness of these materials could have contributed to the relative change in ΔE noted upon immersion of these materials in various food colorants.

The dental literature describes both perceptible and acceptable thresholds of color change in dental composites. Perceptible thresholds are defined as a change in ΔE where the color change is recognized by observers, with most observers recognizing a change in ΔE of 1. Conversely, the threshold at which observers will accept the change is described as the acceptable threshold, with most observers accepting a change in ΔE of 3.7 (23). If these thresholds were to be applied to the changes in ΔE noted in this study, immersion of all the specimens in the food colorant curry would produce unfavorable acceptable thresholds for color change. When considering perceptible thresholds, immersion of all specimens in tea, coffee, and curry, and the immersion of the nanohybrid in wine would produce unfavorable perceptible color changes.

## Conclusions

Within the limitations of this study, the following conclusions can be drawn.

1. All the tested composites exhibited favorable values for water sorption below the ISO upper limit of 40 µg/mm^(3)^; however, the null hypothesis was rejected since there was a statistically significant difference in water sorption values between PermaFlow Pink and Amaris Gingiva.
2. Water immersion produced the smallest relative change in total color for all tested composites.
3. Curry caused the largest relative change in color across all the composite samples, with the pink colored Amaris Gingiva showing the greatest color change
4. Except for all composites placed in the curry colorant and PermaFlo Pink placed in the coffee colorant, the change in total color among the tested composites was below the value of 3.7, or a clinically perceptible difference
5. There was no positive correlation with statistical significance between water sorption and total change in color for any of the composites placed in any of the colorant solutions, but a negative linear relationship between water sorption and color change for PermaFlow Pink immersed in coffee

## Data Availability

The raw data supporting the conclusions of this article will be made available by the authors, without undue reservation.
